# Cardiac regeneration following cryoinjury in the adult zebrafish targets a maturation-specific biomechanical remodeling program

**DOI:** 10.1038/s41598-018-33994-8

**Published:** 2018-10-23

**Authors:** Joseph K. Yu, Padmini Sarathchandra, Adrian Chester, Magdi Yacoub, Thomas Brand, Jonathan T. Butcher

**Affiliations:** 1 Harefield Heart Science Centre, Imperial College London, London, UK; 2 Imperial Centre for Translational and Experimental Medicine, Imperial College London, London, UK; 3 Cornell University, Ithaca, NY USA

## Abstract

Cardiac regeneration post-injury is a tantalizing feature of many lower vertebrates such as fishes and urodeles, but absent in adult humans. Restoration of pumping function is a key endpoint of cardiac regeneration, but very little is known about the biomechanical remodeling process. Here, we quantify and compare the evolution of cellular composition and mechanical stiffness of the zebrafish ventricular myocardium during maturation and following cryoinjury during regeneration to better understand the dynamics of biomechanical remodeling during these two processes. With increasing age, normal myocardial trabecular density and cardiomyocyte fraction increased, while non-myocyte cell fractions decreased. Cell density remained constant during maturation. Cardiomyocyte sarcomeres shortened to a minimum reached at 7.5 months of age, but lengthened with additional age. Concomitantly, ventricular wall stiffness increased up until 7.5 months before plateauing with additional age. Endothelial, myofibroblast/smooth muscle, and cardiomyocyte cell fractions were disrupted following cryoinjury, but were progressively restored to age-specific natural norms by 35 days post infarct (DPI). Infarcted myocardium stiffened immediately following cryoinjury and was a 100-fold greater than non-infarcted tissue by 3 DPI. By 14 DPI, stiffness of the infarcted myocardium had fallen below that of 0 DPI and had completely normalized by 35 DPI. Interestingly, cardiomyocyte sarcomere length increased until 14 DPI, but subsequently shortened to lengths below age-specific natural norms, indicating recovery from a volume overloaded condition. These observations are consistent with the view that regenerating myocardium requires biomechanical stimulation (e.g. strain) to rescue from a volume overloaded condition. Intriguingly, the biomechanical progression of the infarcted adult myocardial wall mirrors that of normal remodeling during aging. The biomechanical progression of the infarcted myocardium targets the values of age-specific norms despite a large divergence in initial conditions. These findings identify a novel biomechanical control of heart regeneration that may orchestrate cellular and tissue level remodeling responses.

## Introduction

The inability of the adult human heart to regenerate myocardium remains a persistent clinical challenge in managing depressed cardiac function and pathological ventricular remodeling following myocardial infarction (MI). Even though cardiomyocytes in the adult mammalian heart do exhibit some renewal^1^, the rate of myocyte proliferation remains insufficient to remuscularize the ventricles and restore cardiac function following MI. Consequently, catheter reperfusion remains the best treatment option by minimizing the size of MI. Despite this intervention, mechanical overload of the heart can still occur which can contribute towards pathological remodeling, additional incidences of MI, and congestive heart failure.

Following MI, dead and dying cardiomyocytes in the infarct are replaced by fibroblasts and excessive collagen, resulting in a stiff non-contractile scar. The mechanical properties of the scar have been shown to be a key determinant of clinical outcome, specifically complications such as infarct rupture, depressed cardiac output, and development of heart failure^2^. However, efforts to improve outcomes through the development of therapeutic interventions aimed at modifying the size and mechanical properties of the post-MI scar have so far yielded mixed results^3^. This is due in part to the fact that the mechanical properties of scar evolve with time—governed by the delicate balance of deposition, degradation, and fibroblast remodeling of the extracellular matrix (ECM), or the protein scaffolding surrounding cells.

Tissue stiffness is in part determined by the ECM and has been shown to be an important developmental cue. The mechanical properties of ECM have been shown to direct differentiation of mesenchymal stem cells into specific lineages^4^ and has also been shown to be important in the maturation of embryonic cardiomyocytes^5^. In the latter, Engler *et al*. demonstrated that pliable substrates resembling the mechanical microenvironment of the developing heart supported myofibril formation and spontaneous beating of embryonic cardiomyocytes. Contrarily, stiff substrates resembling the post-MI scar led to myofibril disassembly and cessation of spontaneous beating. Thus, achieving infiltration and survival of cardiomyocytes in and around stiff scar remains a pressing challenge in obtaining a scar-free regeneration of the ventricles post-MI in the context of cell therapy. Stiff scar tissue is also not an optimal environment to support the viability and maturation of pluripotent stem cell-derived cardiomyocytes^5^. An alternative, unexplored approach is to study the biomechanics of infarct remodeling in an animal model capable of cardiac regeneration. We believe that such an approach can aid in improving cardiomyocyte viability and function following cardiac cell therapy and provide an important comparative biological paradigm.

The adult zebrafish is one such animal that exhibits robust myocardial regeneration following various methods of cardiac injury, including apical resection^6,7^, genetic ablation^8^, and cryoinjury^9,10^. Within two months, the adult zebrafish heart is capable of complete myocardial regeneration following up to 20% resection of the ventricular apex^7^. This regeneration is driven in part by the dedifferentiation and proliferation of resident cortical *gata4*+ and inner trabecular cardiomyocytes^6,11,12^. In response to cardiomyocyte-specific death via targeted genetic ablation, the adult zebrafish heart was capable of complete regeneration following the ablation of over 60% of the ventricular cardiomyocytes^8^. Similarly, regeneration of the ventricular myocardium and restoration of cardiac function was achieved by the dedifferentiation and proliferation of endogenous cardiomyocytes but in only 30 days post-infarct (DPI). Cryoinjury, a technique for inducing localized MI by cooling a region of myocardium to sub-zero centigrade, has been utilized as a method to better recapitulate the inflammation and wound healing process exhibited by human hearts following MI because surgical coronary occlusion is not possible in the adult zebrafish^9,13^.

The biomechanical properties of adult zebrafish ventricular myocardium have not previously been studied. In this study, we quantified and compared changes in the cellular composition and mechanical stiffness of the ventricles during maturation and during regeneration following cryoinjury. We demonstrate that biomechanical remodeling is concomitant in both processes. Following MI, our results suggest that there is an age-dependent homeostatic biomechanical state corresponding to normal maturation and aging that the regenerating myocardial wall targets. While the dynamics of biomechanical remodeling is rather slow during maturation, it is rapid during regeneration.

## Results

### Dynamics of cell composition in the zebrafish ventricular myocardium during maturation

**Figure 1:**
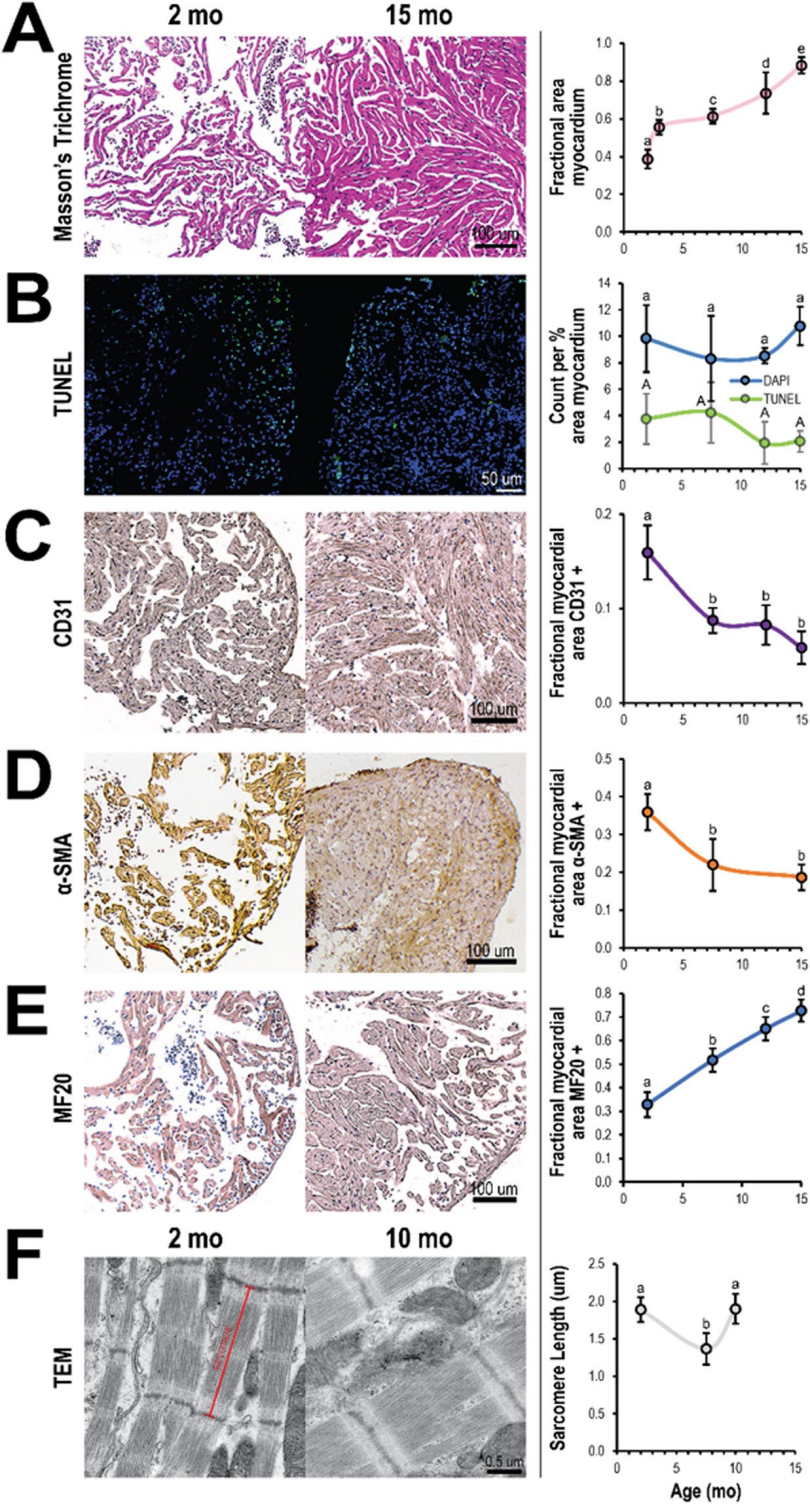
Cellular composition in zebrafish ventricles changes with age. Representative histological sections (left) and quantification (right) of zebrafish ventricles at different ages. (**A**) Ventricles exhibit a gradual increase in myocardial density from 2 to 15 months of age as shown with Masson trichome staining (n ≥ 3) but (**B**) do not show much change in overall cell and apoptotic cell densities (number per percent myocardial area) as shown with DAPI (blue) and TUNEL (green) staining, respectively (n ≥ 4). (**C**–**E**) Ventricles show a significant decrease in fraction of endothelial cells (CD31) (n ≥ 5) (**C**) and myofibroblasts (α-SMA) (n ≥ 5) (**D**) from 2 to 7.5 months of age, but a gradual increase in the fraction of cardiomyocytes (MHC) (n ≥ 6) (**E**). (**F**) Transmission electron microscopy (TEM) of ventricular myocardium reveals sarcomere length was shorter at 7.5 months than at 2 and 10 months of age (n ≥ 16). Significance between different age groups was determined using Tukey’s pairwise test at p < 0.05.

The zebrafish ventricular myocardium exhibited changes in tissue morphology and cellular composition between the ages of 2 and 15 months. Structurally, the density of myocardial trabeculae in the ventricles increased with age (38 ± 5% and 88 ± 4% at 2 and 15 months, respectively) (Fig. 1A). However, the cell density (cell number per percent myocardial area) in the ventricular myocardium did not significantly change nor did the density of apoptotic cells (Fig. 1B). Immunohistochemistry indicated changes in the relative abundance of endothelial cells (CD31+), myofibroblasts and smooth muscle cells (αSMA+), and cardiomyocytes (MF20+) in the ventricular myocardium with age. From 2 and 7.5 months, the fraction of endothelial cells (16 ± 3% and 9 ± 1% at 2 and 7.5 months, respectively) (Fig. 1C) and the fraction of myofibroblasts and smooth muscle cells (36 ± 5% and 22 ± 7% at 2 and 7.5 months, respectively) in the ventricular myocardium fell significantly (Fig. 1D); no significant changes in expression were observed at later time points. Contrarily, the fraction of cardiomyocytes significantly increased (33 ± 5% and 73 ± 5% at 2 and 15 months, respectively) suggesting myocyte proliferation is maintained across the lifespan of the zebrafish (Fig. 1E). Cardiomyocyte sarcomeres shortened with maturation (SL = 1.89 ± 0.16 µm and SL = 1.37 ± 0.21 µm at 2 and 7.5 months, respectively) but then increased with additional age (SL = 1.90 ± 0.20 µm at 10 months) (Fig. 1D).

### Dynamics of cell composition in the zebrafish ventricular myocardium following cryoinjury

**Figure 2:**
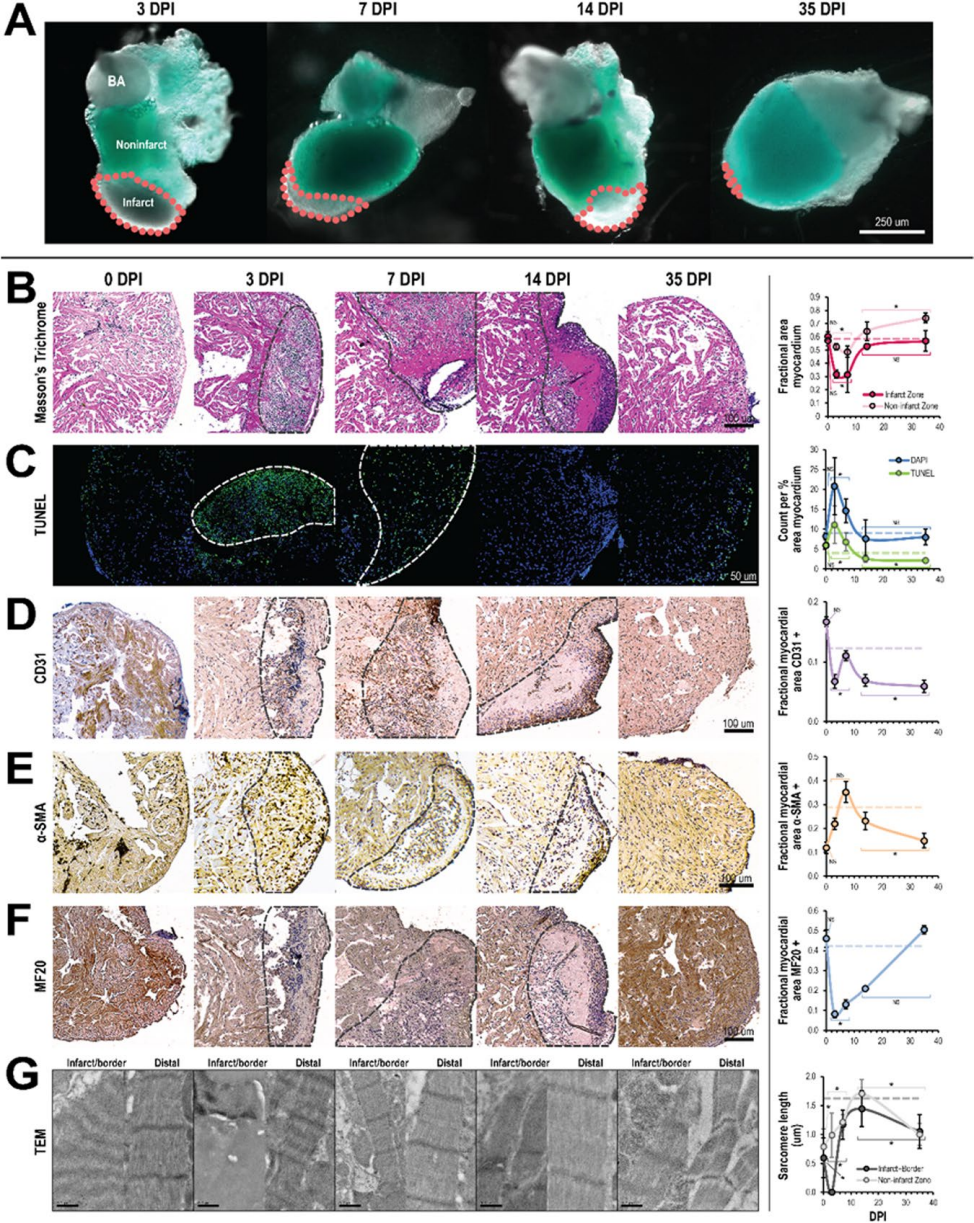
Cellular composition in zebrafish ventricles change following cryoinjury but normalize during regeneration. (**A**) Absence or weak expression of the cmlc2:GFP transgene in the infarcted myocardium. The infarct (gray region, outlined by the dashed lines in pink) progressively diminished in size during the regeneration process and was nearly or completely undetectable by 35 days post infarct (DPI). BA: bulbus arteriosus. (**B**–**G**) Representative histology and TEM images (left) and corresponding quantification (right) of zebrafish ventricles in the days following cryoinjury. For statistical comparison, the biological response to cryoinjury was grouped into three different phases: immediate (0 DPI), remodeling (3 & 7 DPI), and regeneration (14 & 35 DPI). Significant deviations from the extrapolated age-specific baseline mean (dashed horizontal line) was determined Wilcoxon signed-rank test at p < 0.05. (**B**) Masson trichrome staining (n ≥ 6 per phase, 30 total). During the remodeling phase, there was a decrease in myocardium in both the infarct and non-infarct zones. This was subsequently restored during the regeneration phase. (**C**) TUNEL staining (n ≥ 3 per phase, 18 total). During the remodeling phase, overall cell and apoptotic cell densities in the ventricle significantly increased. Cell apoptosis was especially apparent in the infarct zone. In the regeneration phase, overall cell density normalized while apoptotic cell density fell to below the baseline mean. (**D**) CD31 immunostaining (n ≥ 4 per phase, 20 total). The endothelial cell fraction decreased from the immediate to the remodeling phases despite temporarily increasing on 7 DPI; endothelial cell fraction continued to decrease in the regeneration phase. (**E**) SMA immunostaining (n ≥ 3 per phase, 17 total). The myofibroblast fraction rose from the immediate to the remodeling phase, reaching a maximum at 7 DPI before falling in the regeneration phase. (**F**) MF20 immunostaining (n ≥ 4 per phase, 22 total). The cardiomyocyte fraction fell precipitously in the remodeling phase, but normalized during the regeneration phase. (**G**) TEM (n ≥ 15 per time point, 53 total). Significant sarcomere disarray in the infarct and border zone was observed at 0 DPI. No sarcomeres were observed in the infarct zone at 3 DPI but sarcomeres were observed in and around the infarct zone on and after 7 DPI.

The zebrafish ventricular myocardium also exhibited significant changes in tissue ultrastructure and composition during regeneration, especially at the site of cryoinjury. Qualitatively, the infarct zone was distinguishable within 24 hours following cryoinjury because it did not express GFP, indicating local loss of cardiomyocytes (Fig. 2A). Fibrin clots were also observed in some infarcts. By 35 DPI however, the infarct size had significantly decreased and was nearly undetectable. Quantitatively however, the biological response following cryoinjury was grouped into three phases: immediate (0 DPI), remodeling (3 and 7 DPI), and regeneration (14 and 35 DPI). The biological responses in these phases were then compared back to an age-specific baseline mean (4.75 months) extrapolated from the maturation results and significant deviations were determined.

Structurally, the ventricular myocardium exhibited significant changes, especially in the infarct zone (Fig. 2B). During the remodeling phase, the fraction of myocardium in the infarct zone fell drastically to 32 ± 10% (p < 0.001). Concomitantly, significant collagen deposition was observed. Although cardiac muscle fibers were observed in the infarct at 7 and 14 DPI, organized trabecular structure was not observed until 35 DPI (Fig. 2B, not quantified). During the regeneration phase, the fraction of myocardium in the infarct zone had essentially normalized (55 ± 6%, p = 0.15). In the myocardium distal to the infarct, the fraction of myocardium fell during the remodeling phase (50 ± 4%, p < 0.005); subsequently in the regeneration phase, the fraction of myocardium increased (69 ± 7%, p < 0.001) and was significantly higher compared to the baseline mean.

Cell composition was also dynamic in the ventricular myocardium in the weeks following cryoinjury. Compared to the baseline mean, both the total cell and apoptotic cell densities rose significantly during the remodeling phase (18 ± 6 and 8 ± 4, respectively; p < 0.005) (Fig. 2C); apoptosis was primarily localized to the infarct zone (Fig. 2C, left). The rise in total cell density could in part be attributed to the infiltration of immune cells; this was confirmed via TEM (not shown). In the regeneration phase, the total cell density had normalized (8.0 ± 3, p > 0.1) but the apoptotic cell density had fallen below the baseline mean (2.3 ± 0.7, p < 0.05). While the fraction of endothelial cells in the ventricular myocardium (Fig. 2D) was quite high in the immediate phase (17 ± 0.8%, p > 0.05), it fell during the remodeling phase (8 ± 2%, p < 0.005); despite this, endothelial cells could be observed accumulating along the edges and infiltrating the infarct, especially at 7 and 14 DPI (Fig. 2C, left). In the regeneration phase, the overall fraction of endothelial cells remained less than the baseline mean (6 ± 1%, p < 0.005). The fraction of myofibroblasts/smooth muscle cells in the ventricular myocardium gradually increased in the days following cryoinjury (Fig. 2E); this was especially apparent in the regenerating infarcted myocardium. Growing from 12 ± 2% in the immediate phase to 26 ± 7% in the remodeling phase, the fraction of myofibroblasts/smooth muscle cells subsequently falling to 20 ± 5% in the regeneration phase; compared to the baseline mean, only the regeneration phase deviated significantly (p < 0.01). The fraction of cardiomyocytes in the ventricular myocardium (Fig. 2F) fell precipitously between the immediate (46 ± 4%, p > 0.1) and remodeling (11 ± 3%, p < 0.001) phases; loss of cardiomyocytes was especially evident in the infarct zone. In the regeneration phase, the fraction of cardiomyocytes was rose significantly and rapidly normalized (36 ± 17%, p > 0.10).

Within cardiomyocytes in the infarct and border zone surrounding the infarct, we observed that sarcomeres decreased in length (0.597 μm, p < 0.0001) and lost their typical distinct, dark Z-lines in the immediate phase following cryoinjury (Fig. 2G). In the remodeling phase, no sarcomeres were observed in the infarct and border zones at 3 DPI; by 7 DPI however, sarcomeres with distinct Z-lines were observed. Compared to the baseline mean, cardiomyocyte sarcomere length in the infarct and border zone in the remodeling phase was significantly shorter (1.084 ± 0.339 μm, p < 0.0001). In the regeneration phase, sarcomeres in the infarct and border zones were shorter compared to the baseline mean (1.297 ± 0.356 μm, p < 0.0001). Within cardiomyocytes distal to the infarct, the average sarcomere length remained shorter compared to the baseline mean (p < 0.0001) despite sarcomere length elongation in the remodeling and regeneration phases. Sarcomere length in the immediate phase rose from 0.789 ± 0.306 μm (p < 0.0001) to 1.165 ± 0.232 μm (p < 0.0001) and 1.196 ± 0.377 μm in the remodeling and regeneration phases, respectively. Within the regeneration phase however, sarcomeres throughout the ventricles were observed to shorten between 14 and 35 DPI.

### Dynamics of mechanical stiffness of the zebrafish ventricular myocardium during maturation and regeneration following cryoinjury

**Figure 3:**
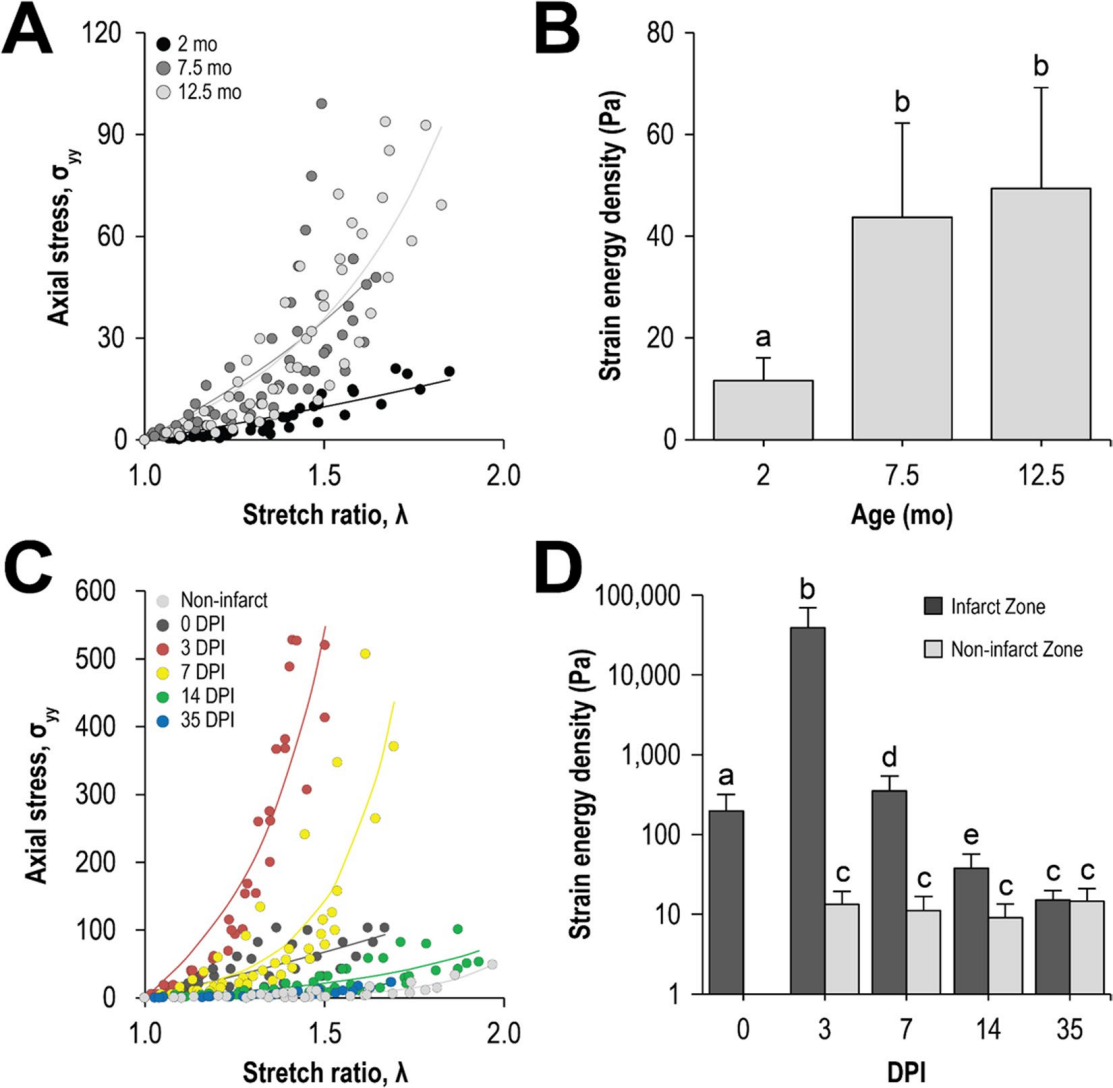
The zebrafish ventricular myocardium stiffens during maturation and following cryoinjury but softens during regeneration. Representative pressure change versus stretch ratio curves of (**A**) maturing and (**C**) regenerating zebrafish ventricular myocardium. Strain energy density, a metric for tissue stiffness, was extracted from these curves and plotted for the (**B**) maturation and (**D**) regeneration processes. (**B**) The ventricle in the maturing zebrafish significantly stiffened between the ages of 2 and 7.5 months, which corresponds to the transition between juvenile to adult state (n ≥ 4). (**D**) The infarct zone significantly stiffened following cryoinjury at 0 DPI and continued to stiffen do so until 3 DPI. Afterwards, the infarct zone softened and by 35 DPI had normalized. Away from the infarct zone, the stiffness of the ventricular myocardium did not change (n ≥ 6).

The mechanical stiffness of the ventricular myocardial wall also changed with concomitant structural and compositional changes during maturation and regeneration following cryoinjury. The ventricular myocardium stiffened with age and exhibited significant stiffening between the ages of 2 and 7.5 months (11.6 ± 4.5 Pa and 43.7 ± 1  Pa, respectively; p < 0.05) (Fig. 3A,B). Following cryoinjury, the infarcted myocardium stiffened more than two orders of magnitude (Fig. 3C,D); the stiffness of the infarct zone was 196 ± 12  Pa at 0 DPI, rose to and reached a maximum of 38.9 ± 30.6 kPa at 3 DPI, before falling at later time points and normalizing by 35 DPI (15 $\pm$ Pa). Away from the infarct, the ventricular myocardium exhibited no significant changes in mechanical stiffness (~13 Pa).

### Comparison of biomechanical states of the zebrafish ventricular myocardium during maturation and regeneration

**Figure 4:**
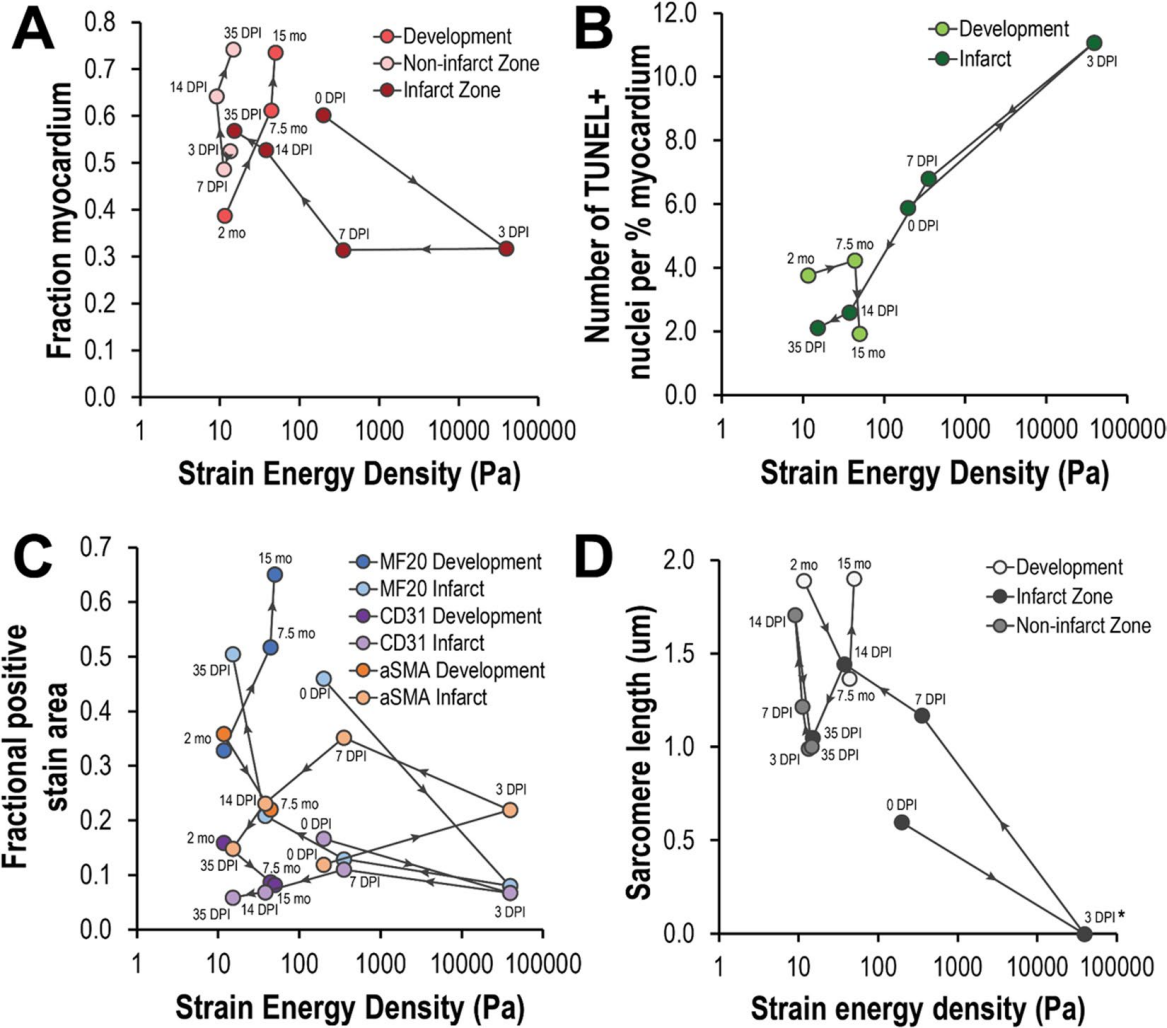
The zebrafish ventricular myocardium seeks out age-specific biomechanical homeostasis. Biomechanical correlations of zebrafish ventricular myocardium dynamics are shown across maturation and regeneration. Each set of points along a given line indicates measurements taken during maturation or during regeneration. The arrows between points indicate the progression of time. (**A**) Compared to maturation, the ventricular myocardium during regeneration was less stiff despite having a larger fraction myocardial area. While the fraction of myocardial area away from the infarct at 35 DPI is comparable to the fractional myocardial area at 15 months of age, the fraction of myocardial area at the site of infarct is more comparable to that of 7.5 months of age. (**B**) Following initial inflammation and stiffening of the infarct at 3 DPI, the apoptotic cell density drastically fell and approached basal levels by 14 DPI comparable to that of 15 months of age. (**C**) While the cardiomyocyte (MF20+) fraction in the ventricle rose during maturation, it fell precipitously following cryoinjury but began to recover after 3 DPI; by 35 DPI, the fraction of cardiomyocytes was comparable to that of hearts in zebrafish 7.5 months of age. The endothelial cell fraction during maturation were comparable to that of regeneration. Revascularization of the ventricles following cryoinjury was most notable at 7 DPI, where the endothelial cell fraction peaked during the healing and regeneration process. (**D**) On average, sarcomere length was longer in cardiomyocytes in the developing zebrafish heart as opposed to the healing and regenerating zebrafish heart. Sarcomere length was observed to elongate in cardiomyocytes both in and away from the infarct between 7 and 14 DPI but was observed to contract between 14 and 35 DPI.

To better compare the progression of biomechanical states of the ventricular myocardium during maturation and regeneration, cell quantification and mechanical stiffness data were plotted together for both processes in Fig. 4; levels of expression are plotted against myocardial stiffness and temporal evolution is labeled. In doing so, we can better visualize the biomechanical progression, or the dynamics of biological and mechanical tissue states across time. While maturation progressed along one curve with increasing age, regeneration progressed along another, deviating from this natural progression but ultimately returning to the appropriate age-specific homeostatic point by 35 DPI. The biomechanical progression of myocardial trabecular area for the non-infarcted myocardium during regeneration was very similar to that of the maturing myocardium (Fig. 4A) having a left and upward trajectory with time. Contrastingly, biomechanical progression of myocardial trabecular area for the infarcted myocardium demonstrated an initial down and rightward trajectory concomitant with stiffening and loss of viable myocardium; recovery of myocardial trabecular area did not begin to occur until after 7 DPI or when mechanical stiffness was below ~350 Pa. While biomechanical progression of apoptotic cell density (Fig. 4B) remained relatively static during maturation, its trajectory following cryoinjury acutely moved upwards and to the right between 0 and 3 DPI before progressing in the exact opposite direction and essentially normalizing by 14 DPI. The biomechanical progression of cardiomyocyte, endothelial cell, and myofibroblasts/smooth muscle cell fractions are summarized in Fig. 4C. The biomechanical progression of cardiomyocyte cell fraction during maturation and regeneration mirrored that of myocardial trabecular area (Fig. 4A). The biomechanical progression of endothelial cell fraction had a slight down and rightward trajectory. Following cryoinjury, the biomechanical progression of endothelial cell fraction acutely deviated significantly to the right and only slightly downwards; its trajectory was primarily leftward after 3 DPI with a slight initial upward progression as well. The biomechanical progression of myofibroblasts/smooth muscle had a right and downward trajectory; following cryoinjury however, its trajectory was significantly more dynamic, first demonstrating a right and upwards trajectory before circling back and normalizing towards the lower left quadrant. While the biomechanical progression of sarcomere length (Fig. 4D) for the non-infarcted myocardium during regeneration was essentially opposite to that of the maturing myocardium, their states were comparable. The biomechanical progression of sarcomere length deviated significantly down and to the right between 0 and 3 DPI before moving upwards and to the right afterwards until 14 DPI. By 35 DPI, sarcomere length states in the formerly infarcted myocardium was equivalent to that in non-infarcted myocardium.

## Discussion

The bulk stiffness of the ventricular myocardium can be attributed to the stiffness of the individual tissue components, tissue composition, and degree of mechanical coupling; specifically, they include stiffness of individuals cells and ECM protein structures, cell and ECM composition, cell-cell and cell-ECM mechanical coupling and ECM cross-linking. Changes in any of these components can lead to changes in the mechanical stiffness of the ventricular myocardium. Our characterization of the dynamics of cellular composition, cardiomyocyte sarcomeres, and mechanical stiffness of the ventricular myocardium in the zebrafish across maturation and regeneration attempts to characterize the biological dynamics underlying changes in mechanical stiffness of the ventricular myocardium. We also sought to reveal parallels between the processes of maturation and regeneration following cryoinjury and provide a foundation for subsequent investigations on the role of biological and mechanical stimuli in both processes.

During maturation, we observed a progressive increase in the trabecular density of the ventricular myocardium; however, myocardial stiffness only significantly rose between the ages of 2 and 7.5 months, which corresponds to the transition between juvenile and adulthood^14^. While the trabecular density increased with age, the overall myocardial cell density did not change. This arose from changes in cell composition within the ventricular myocardium. While the fraction of endothelial cells, myofibroblasts, and smooth muscle cells decreased with age, the fraction of cardiomyocytes increased. This could arise from continued cardiomyocyte proliferation, cardiomyocyte hypertrophy, or both. The combination of the two would enhance the ability of the ventricle to pump blood. The latter would contribute toward increasing cardiomyocyte stiffness^15^. In the developing embryonic zebrafish heart, thickening of the ventricular myocardium and cardiomyocyte hypertrophy correlate to rising arterial blood pressure and cardiac demand^16^. The same mechanical forces could play an important role in ventricular maturation, especially during the juvenile to adult transition where the zebrafish reaches maximal physical stature. Additional changes in ECM state and gradual remodeling could contribute towards subsequent stiffening of the ventricular myocardium with increasing age during adulthood^17^.

Following cryoinjury, we observed significant stiffening of the infarcted myocardium. This initial stiffening at 0 DPI in the immediate phase can be attributed to myosin cross-bridge fixation in infarcted cardiomyocytes resulting in impaired diastolic relaxation. In the early remodeling phase at 3 DPI, the infarct was nearly three orders of magnitude stiffer than non-injured myocardium. Extensive apoptosis and fibrosis in the infarcted are was also observed. The significant increase in stiffness can be attributed to extensive fibrosis at the infarct site as the ECM is the primary component maintaining the integrity of the infarcted myocardium, as well as infiltration from immune cells. No cardiomyocyte sarcomeres were visible in the infarct via TEM. In the late remodeling phase at 7 DPI, revascularization and remuscularization of the infarcted ventricular myocardium was observed to begin. Sarcomeres were observed in the infarct site. Remodeling and removal of the fibrotic scar by myofibroblasts was underway, as indicated by maximal expression of αSMA and a noticeable decrease in infarct size. In the early regeneration phase at 14 DPI, there was significantly less collagen at the infarct site. In the late regeneration phase at 35 DPI, the normalization of ventricular myocardial stiffness was concomitant with normalization of cell composition and restoration of trabecular structure. There were no significant changes in stiffness of the ventricular myocardium away from the infarct site.

Sarcomere length, an indicator of cardiomyocyte contractile capacity, was observed to both elongate and shorten during maturation and regeneration. The force-length or Frank-Starling relationship dictates that the force of contraction is dependent on the stretched length of cardiac muscle fibers or sarcomere length. It has been shown in a murine model that the stiffening of the ventricular myocardium with age can be partly attributed to stiffening of individual cardiomyocytes and that this stiffening impairs lengthening of the cardiomyocyte sarcomere during ventricular filling^18^. Changes in sarcomere length could indicate changes in force-length relationships across maturation, aging, and regeneration. We propose that cardiomyocytes in the maturing ventricle would tend towards a state of high pumping efficiency at the cost of limited robustness (i.e., hypertrophied with well-developed sarcomeres, with optimized stretch and force generation across various preload conditions), while cardiomyocytes in the developing ventricle would tend towards a state of high robustness (i.e., ability to proliferate) but at the cost of limited force development (i.e., less organized sarcomeres, less optimal stretch and force generation across various preloads). Elongation of sarcomeres with additional aging in adulthood could indicate the existence of an optimal age where the heart reaches the peak of cardiac contractile performance, in which sarcomeres are stretched to the optimal lengths for a given diastolic relaxation cycle. Of course, cardiac pumping efficiency and peak performance is relative to the metabolic state of the fish or, in this case, at rest. Additional work is required to elucidate the changes in cardiomyocyte stiffness and force-length relationship during zebrafish maturation and aging.

In mammals, the vicious cycle of volume overload leads to impaired cardiomyocyte contractility following MI which contributes to myocardial remodeling and the onset of dilated cardiomyopathy. However, this pathogenic response is successfully counteracted in the zebrafish following regeneration. The exact mechanisms remain unknown however, specifically how and when dedifferentiated and proliferating cardiomyocytes begin to contribute towards cardiac contractions. De novo cardiomyocytes in the infarct zone and cardiomyocytes in the non-infarct zone demonstrated initial elongation of sarcomeres under volume overloaded conditions (3–14 DPI) but shortening upon full regeneration (14–35 DPI). Considering that the fibrotic scar is stiff, this suggests that dedifferentiated cardiomyocytes in the infarct zone do not contribute much to the overall cardiac contraction but can be called upon for greater contraction in the case of significant volume overload. Future work should further delve into characterizing dynamics of force production in proliferating dedifferentiated cardiomyocytes during regeneration, in the context of fibrotic scar remodeling, volume overload, and hypoxia.

Our results demonstrating restoration of cellular composition in the ventricular myocardium aligns with previous published results indicating recovery of ventricular function and hemodynamics following cardiac regeneration^19,20^. However, localized wall movement abnormalities were observed to persist up to 180 DPI and that this corresponded to persistent localized structural alterations in the cortical layer following regeneration^19,21^. This indicates that both restoration of cellular composition and myocardial stiffness may not fully reflect restoration of contractile ability. Specifically, dedifferentiated cardiomyocytes might require additional time to mature; shortened sarcomeres at 35 DPI suggest a less mature phenotype on average throughout the ventricle. While unexplored in our study, regeneration and restoration of the trabecular architecture, an important structural component of the contracting heart, would probably occur secondarily to the initial restoration of cellular composition and cardiomyocyte sarcomeric structure. Perturbations in local wall stresses and its effects on restoration of trabecular architecture could also help to better understand the role of mechanical stimulation in this process.

This is particularly interesting in light of the recent discovery of a regulatory element in the connective tissue growth factor a (*ctgfa*) gene termed *careg*, during cardiac regeneration following cryoinjury^12^. The same *careg* element is activated both during embryonic cardiac development and fin regeneration. During regeneration, *careg-expressing* dedifferentiating cardiomyocytes in the peri-injury zone contribute towards the formation of new myocardium; *careg*-dependent expression of *ctgfa* was dependent on TGFβ/Activin-β signaling. *Pfefferli & Jazwinska* proposed that *careg* element acted as a biosensor triggered by TGFβ/Activin-β in regenerating cells. It would be interesting to delve into the biophysical mechanisms of *careg* activation, the effect of volume and pressure perturbations on it, and exploring its role in the dynamics of scar remodeling.

While pipette aspiration as a methodology for quantifying local myocardial stiffness is quite robust, it however lacks additional spatial resolution. While local, nonlinear stiffness can be measured via strain energy, uniaxial stress via pipette suction lacks the ability to resolve potential anisotropy of stiffness. Additionally, thinning of the myocardium or detachment of the epicardium could lead to underestimation of tissue stiffness due to global tissue deformation contributing to perceived local tissue deformation.

Altogether, we identified a natural ventricular myocardial stiffness threshold achieved by the adult zebrafish. We further identify a marked stiffening of the ventricular myocardium following infarction that is progressively relieved during regenerative remodeling. Additionally, our findings recapitulated important changes in myocardial wall composition and structure, thereby confirming the progression of inflammation, fibrosis, healing, and regeneration following cryoinjury. Lastly, we showed how the biomechanical progression of regeneration seeks to restore the age-dependent biomechanical homeostasis but from a different and accelerated trajectory.

## Methods

Zebrafish husbandry and all experiments were carried out in accordance with the United Kingdom (UK) Animal (Scientific Procedures) Act 1986 and approved by the UK Home Office.

### Cryoinjury

Adult zebrafish (4–6 months old, cmlc2:GFP) were first anaesthetized in a bath of 0.032% (wt/vol) Tricaine and then secured ventral side up in a foam holder dampened with system water. Ventral scales between the gills were removed with forceps and a transverse incision was made through the skin and pericardium to visualize the ventricle. Using a tissue, excess moisture was removed from the surface of the ventricle. Occasionally, gentle pressure was applied to the abdomen to better visualize the ventricle. A liquid nitrogen-cooled cryoprobe was gently applied to the ventricular apex to induce MI with size ~20% of the ventricular volume. Successful contact would lead to formation of ice crystals around the probe and ventricle, causing the two to adhere. The probe was then removed once the ice crystals thawed, usually within 10–15 seconds. Fish were then placed into a bath of warm system water, allowed to revive, and then monitored for the appropriate duration before sacrifice. The procedure was well tolerated by animals (>90% survival); nearly all zebrafish survived if they were revived.

### Micropipette Aspiration

Mechanical testing via micropipette aspiration was performed as described previously^22^. Briefly, glass micropipettes were pulled from borosilicate glass capillary tubes using a P-30 vertical pipette puller (Sutter) to an inner diameter of 90–120 µm; jagged tips were smoothed over a flame. Micropipettes were bent (~30°) over a flame. Isolated hearts were immersed in 4 °C PBS and stored on ice until mechanical testing. Hearts were placed in a clear Petri dish with chilled PBS and visualized under a scope when ready for testing. Using a micromanipulator, the pulled tip of a glass micropipette was positioned along the epicardial surface of the ventricle at the site of interest. The other end of the glass micropipette was connected to an air displacement micropipette (Oxford) with rubber tubing. Incremental negative pressure was applied to the ventricular myocardial wall by twisting the thumb knob and increasing the micropipette volume. The deformation of the myocardial wall into the glass micropipette was imaged and then measured to extract a stretch ratio vs. pressure change curve. Curves were fitted to an equation describing axial Cauchy stress for a uniaxial load of an incompressible material with an assumed exponential material law; strain energy density was then extracted to describe myocardial wall stiffness. A minimum of 4 specimen per treatment condition were analyzed, and significant changes were identified by ANOVA and post-hoc testing (P < 0.05, Tukey HSD pairwise comparison).

### Immunohistochemical staining and TUNEL assay

Immunohistochemical staining was used to visualize tissue structure; quantify expression levels of markers for endothelial (1:25, CD31 rabbit, Abcam), myofibroblasts (1:200, αSMA mouse, DAKO), and mature cardiomyocytes (1:10, MF20 mouse, Developmental Studies Hybridoma Bank); and identify apoptotic cells (TUNEL assay). Zebrafish hearts were fixed in 10% formal saline, processed into paraffin blocks, sectioned (5μm), and then mounted onto glass slides. Mounted samples were then dewaxed; rehydrated in water; washed in phosphate buffered saline (PBS) (5 min); immersed in 0.1 M citrate buffer (pH 6) and microwaved (10 min); and blocked for endogenous peroxidases using 0.3% hydrogen peroxide in PBS (10 min). Sections were washed 3X in PBS (5 min each), blocked with 3% (w/v) bovine serum albumin (BSA) in PBS (30 min), and then incubated with primary antibodies overnight. Subsequently, sections were then washed 3X in PBS; incubated in biotinylated goat anti-rabbit immunoglobulins for CD31 and mouse IgG for MF20 (1:250, Vector Laboratories) for 1 hour; washed 3X in PBS; and then incubated with Avidin-Biotin Complex (ABC-Vector Laboratories) for another hour. Reactivity was detected using diaminobenzidine tetrahydrochloride (DAB tablets- Sigma) (25 mg/ml) and hydrogen peroxide (0.01% W/V). Sections were then counter stained with haematoxylin and viewed on Ziess Axioskop microscope. For TUNEL, sample preparation and incubation with TUNEL labelling solution (ROCHE) was carried out according to the manufacturer instructions.

Images were analyzed using ImageJ. Appropriate thresholding was applied to identify positively and negatively stained regions for immunohistochemistry samples. Percent marker expression was calculated as the area of positively stained tissue divided by the total myocardial area. Total cell count and apoptotic cell counts were extracted from images obtained from samples stained with DAPI and TUNEL.

### Transmission Electron Microscopy (TEM)

Samples of zebrafish ventricles were imaged via TEM to visualize sarcomere structure and quantify sarcomere length. Zebrafish hearts were fixed in 2.5% (v/v) glutaraldehyde in 0.1 M sodium cacodylate buffer (pH 7.4) at 4 °C overnight and postfixed in 1% (w/v) osmium tetroxide in 0.1 M sodium cacodylate buffer at room temperature for 1 hour. After 2 buffer washes, heart samples were dehydrated using a graded ethanol series of 50%, 70%, 90% and 100% (v/v), immersed in propylene oxide, and infiltrated with 1:1 propylene oxide:Araldite resin (Agar Scientific) overnight. Samples were then twice immersed in baths of pure resin for two hours each, embedded, and incubated at 60 °C for 24 hours to polymerize the resin. 100 nm thin sections were then cut with an ultramicrotome and mounted on 300 mesh copper TEM grids; Grids were stained using uranyl acetate and lead citrate and imaged on a JEOL 1200 EX microscope. Sarcomere length was measured as the distance between adjacent Z-lines.

### Quantification of histological changes and statistical analysis

Immunohistochemistry and TEM (n ≥ 3 independent samples per time point for maturation; n ≥ 3 for grouped phases for regeneration following cryoinjury) and mechanical testing data (n ≥ 3) are reported as means with error bars represent standard deviation from the mean. In maturation and mechanical testing data sets, statistical significance between time points was determined using Tukey’s post hoc paired tests for treatment effects. In the regeneration data set, statistical significance between phases (i.e., response-shared grouped time points) and the age-specific baseline mean (4.75 months) was determined using Wilcoxon signed-rank test followed by a post hoc one sample t-test; the mean was extrapolated from the maturation data. Differences were considered significant for p < 0.05.
